# Comparison of the Effects of Eicosapentaenoic Acid and Docosahexaenoic Acid on the Eradication of *Helicobacter pylori* Infection, Serum Inflammatory Factors and Total Antioxidant Capacity

**Published:** 2015

**Authors:** Nafiseh Khandouzi, Farzad Shidfar, Shahram Agah, Agha Fatemeh Hosseini, Afsaneh Dehnad

**Affiliations:** a *Department of Nutrition and Biochemistry, School of Nutritional Sciences and Dietetics, Tehran University of Medical Sciences, Tehran, Iran. *; b *Department of Nutrition, School of Public Health, Iran University of Medical Sciences, Tehran, Iran. *; c *Colorectal Research Center, Iran University of Medical Sciences, Tehran, Iran. *; d *Department of Math and Statistics, School of Health Management and Information Sciences, Iran University of Medical Sciences, Tehran, Iran. *; e *Department of Foreign Languages, School ofHealth Management and Information Sciences, Iran University of Medical Sciences, Tehran, Iran.*

**Keywords:** Helicobacter pylori, Eicosapentaenoic acid, Docosahexaenoic acid, Eradication, Inflammatory factors

## Abstract

Helicobacter pylori infection, the most common chronic bacterial infection in the world, and an important cause of gastrointestinal disorders, may be involved in the pathogenesis of some extra-gastrointestinal disturbances, as well as an increase in blood levels of certain inflammatory markers. Anti-bacterial activity against Helicobacter pylori and anti-inflammatory properties of omega-3 fatty acids have been studied in several research studies. The purpose of the present study was the comparison of the effects of Eicosapentaenoic Acid and Docosahexaenoic Acid supplementation on Helicobacter pylori eradication, serum levels of some inflammatory markers and total antioxidant capacity. In a randomized, double-blind, placebo-controlled clinical trial, 97 Helicobacter pylori positive patients (64 patients in the two intervention groups and 33 in the control group), received 2 grams daily of Eicosapentaenoic Acid, Docosahexaenoic Acid or Medium Chain Triglyceride oil as placebo, along with conventional tetra-drug Helicobacter pylori eradication regimen, for 12 weeks. Helicobacter pylori eradication test and measurement of concentration of interleukine-6, interleukine-8, high-sensitivity C-reactive protein and total antioxidant capacity were performed after the intervention. There was no significant difference in eradication rate of the infection, levels of interleukine-6 and total antioxidant capacity among the three groups, while the levels of interleukine-8 and high-sensitivity C-reactive protein were statistically different. Eicosapentaenoic Acid or Docosahexaenoic Acid supplementation had no significant differential impact on the eradication of Helicobacter pylori infection, and serum levels of interleukine-6 and total antioxidant capacity. However, it had a desirable effect on the levels of interleukine-8 and high-sensitivity C-reactive protein in Helicobacter pylori positive patients.

## Introduction

Helicobacter pylori (H. pylori) infection, the most common chronic bacterial infection in the world (1), is the main etiologic factor of peptic ulcer and is responsible for several gastrointestinal disorders (2). It is the first bacterium known as definite carcinogen by the World Health Organization's International Agency for Research on Cancer (3). While about half of the world population is reported to be the carriers of this organism (4), the prevalence of the infection among the adult population in the Middle East and Iran has been reported 70%-90% (2) and 69% (5), respectively.

Complications of the infection depend on the complex interactions between the bacteria and the host, such as infection virulence, genetic sequence, the age of the host, environmental factors and dietary habits (6). Several studies have suggested that H. pylori infection may be involved in the pathogenesis of some extra-gastrointestinal disturbances. This bacterium with the effects such as endothelial injury, smooth muscle proliferation, local inflammation of blood vessel walls (1), and the effect on lipid metabolism (1,2,7) is considered as a risk factor for cardiovascular diseases (8). The relationship between this bacterium and atherosclerotic diseases, gallbladder stone, dyspepsia, metabolic syndrome, type 1 diabetes mellitus and insulin resistance has been observed in a number of studies conducted in Iran (9).

Many studies have shown that H. pylori infection elevated production of pro-inflammatory cytokines, regulators of immune and some peptide chemokines such as interleukin-1β (IL-1β) (10), interleukin-6 (IL-6) (11), interleukin-8 (IL-8) (12), interleukin-12 (IL-12) (13), tumor necrosis factor-α (TNF-α) (14), interferon-γ (INF-γ) (15) and high-sensitivity C-reactive protein (hs-CRP) (16) in epithelial cells of the stomach. 

Omega-3 fatty acids (n-3 FAs) have shown to be beneficial due to their anti-inflammatory, anti-thrombotic, anti-arrhythmic, hypolipidemic and vasodilatory properties (17). N-3 FAs, by blocking the production of eicosanoids, derived from arachidonic acid, show anti-inflammatory actions (18). In addition to decreasing the production of leukotriene B4, thromboxane A2, prostaglandine E1, interleukin-1, interleukin-6 and tumor necrosis factor, n-3 FAs neutralize free radicals by reducing the amount of arachidonic acid (17). Cell culture studies have shown that major n-3 FAs, Eicosapentaenoic acid (EPA) and Docosahexaenoic acid (DHA) can also inhibit the production of inflammatory cytokines such as TNF-α, IL-1β, IL-6 and IL-8 by monocytes, macrophages and endothelial cells (18). 

There are a number of studies suggesting that EPA and DHA have anti-bacterial activity against H. pylori in some conditions (19-21). Furthermore, various studies have shown that EPA and DHA have different clinical effects on gene expression (22), blood pressure (23,24), vascular function (24), serum lipid profile (25) like triglycerides (26) and HDL-C (24), lipoprotein particles size like HDL-C, LDL-C and VDLD (24,26), serum glucose and insulin in hyperlipidemic male adults (25), inflammatory mediators (27-29), mood disorders-depression (30,31), visual disorders (32-35), and cognitive disorders-dementia (36).

Due to the high prevalence of H. pylori infection, consequences of untreated chronic infection, and that to our knowledge, no existing human research has addressed differential effects of supplementation with long chain omega -3 fatty acids (EPA and DHA), on the eradication of this infection and the levels of some serum inflammatory markers in H. pylori positive patients, the present study was designed to compare the effects of EPA and DHA supplementation on H. pylori eradication and the serum levels of inflammatory markers and total antioxidant capacity.

## Experimental

*Materials and methods*

The present study was a randomized, double-blind, placebo-controlled clinical trial. The population of the study consisted of all adult patients, aged 20-60, who were admitted to the Gastrointestinal and Liver Disease Research Center of Tehran University of Medical Sciences (Rasool Akram Hospital). The under study patients were those who met the criteria of the study and diagnosed with H. pylori infection by a gastroenterologist on the basis of the pathological results of the stomach biopsy. The criteria of the study were: no history of treatment for H. pylori eradication, no stomach surgery, no history of peptic ulcer, no diseases causing inflammation or increased levels of inflammatory markers such as diabetes mellitus, no cardiovascular diseases, no pulmonary inflammations such as asthma, *etc*. Other criteria were: taking no medications other than those prescribed during the study period, taking no antibiotics or bismuth during the previous 2 months, taking no antioxidant supplements such as selenium, zinc and beta-carotene for at least 3 months prior to the study, taking no omega-3 supplements for at least 3 months prior to the study, no smoking, and having a BMI of less than 30. Exclusion criteria of the study were: changes in the type or dose of medication, changes in diet or daily physical activity, any use of antioxidant supplements and consumption of less than 80% of supplements during the study period.

The sample size was determined on the basis of data from previous studies (19,37,38) by considering type I error of 0.05 with the test power of 80%. To insure that the sample size, used in the study, could obtain the desired outcome for all relevant variables, the highest number of the calculated was considered. The sample size was computed as 30 per group. Regarding a possible loss in the follow-up, a safety margin of 15% was determined, therefore, 35 patients were assigned to each group.

After attending the orientation session and filling out the written informed consent, the participants were enrolled, and 105 eligible patients of either sex included in the study, and randomly allocated in three groups. 

At the beginning of the study, information on socio-economic status of participants was collected by means of a checklist completed through an interview. For each person, while wearing light clothes, body weight was measured, using a SECA scale with the precision of 0.5 Kg. Each person’s height was measured in standing position without shoes, using a tape meter with precision of 0.5 cm. Finally, Body Mass Index (BMI) was calculated in the form of weight in kilograms divided by height in meters squared [weight (Kg)/height (m)^2^]. At the beginning of the study, 10 ^cc^ blood samples were obtained from patients after 10-12 hours of fasting.

The report of daily dietary intake was collected by a 24-hour diet recall questionnaire once before the beginning of the study, then in week 4, week 8 and finally at the end of the study. The data was then analyzed by Nutritionist IV software. The reports of the level of physical activity, at the beginning and the end of the study, were obtained through an interview with each participant, using the International Physical Activity Questionnaire (IPAQ). Patients were asked not to change their usual dietary intake and physical activity during the study.

Participants were randomly assigned to EPA, DHA or control groups, receiving 2 g daily morEPA, morDHA or placebo for 12 weeks, respectively. Each morEPA and morDHA supplement had a purity of 75% containing 750 mg of n-3 FA, produced by Minami-nutrition Company in Belgium. MorEPA contained 580 mg EPA, 83 mg DHA and 87 mg other n-3 FAs, and morDHA contained 63 mg EPA, 465 mg DHA and 222 mg other n-3 FAs. The placebo, used in the study, was Medium Chain Triglyceride (MCT) oil, in 1 g capsules with a purity of 100%, specifically designed by Canadian company of Viva to resemble omega-3 capsules. Before the beginning of the study, all tins containing the capsules were coded by someone other than the researchers. Patients, in all three groups, received conventional tetra-drug H. pylori infection eradication regimen for 12 weeks. This drug protocol includes metronidazole (500 mg twice a day), amoxicillin (1 g twice a day), bismuth subcitrate (240 mg twice a day), and omeprazole (20 mg twice a day).

During the 12-week intervention practice, the patients were called to resolve possible problems and ensure that the participants had consumed the drugs and supplements. At the end of 12^th^ week, the patients were asked to refer for H. pylori eradication check and related blood tests. All initial checks were repeated except height measurement. 

Blood samples were collected into clean and dry test-tubes without EDTA and the serum was separated by centrifugation (3000 rpm at 4 ^°^C for 10 min) and stored at -80^°^C for biochemical analysis. Serum levels were calculated in the following ways: IL-6 and IL-8 by ELISA method using Bendermed company kit from Austria and hs-CRP by ELISA method using IBL company kit from Germany. ELISA (Enzyme-linked immunosorbent assay) is a simple, effective assay platform used in the quantitative measurement of either secreted or intracellular protein levels in biological samples such as cell lysates or serum. ELISAs have established themselves as the gold standard for protein quantitation over other platforms for their intra- and inter-assay variability, ease of use and sensitivity. The ELISA platform provides a specific, sensitive detection of protein levels in non-purified samples. All of this allow for the accommodation of a large number of samples, in a fast and inexpensive platform (39).

TAC was measured by colorimetric method (FRAP) using TPTZ (2,4,6-tri[2-pyridyl]-1,3,5-triazine) indicator of Sigma Company from U.S.A. Fluorescence Recovery After Photobleaching (FRAP) is the method to determine antioxidant capacity in extracts (40). FRAP method, rapidly applied, was simple highly and reproducible. FRAP assay depends upon the ferric tripyridyltriazine (Fe (III)-TPTZ) complex to the ferrous tripyridyltriazine (Fe (II)-TPTZ) by a reductant at low pH. Fe (II)-TPTZ has an intensive blue color and can be monitored at 593 nm (41). 

Data entry and statistical analysis were performed by using SPSS_16_ software. Quantitative variables were reported with mean and standard deviation (as Mean ± SD) and their normal distribution was determined by Kolmogorov-Smirnov test. To compare the infection eradication rate between the three groups, a chi-square test was performed. The difference in blood biochemical variables was compared, before and after the intervention, in each group. For variables with normal distribution, a paired t-test and for variables other than those, the wilcoxon test was used. To compare the quantitative variables, normal and non-normal distribution, between the three groups, Analysis of Variance (ANOVA) and Kruskal-wallis tests were used, respectively. Differences with p<0.05 were considered significant. 

The protocol of the study was in compliance with the Helsinki Declaration of 1975 (revised in 2008) and informed consent form was obtained from each patient. This study was approved by the Ethics Committee of Public Health School of Tehran University of Medical Sciences and registered in Iranian center of clinical trial registration with the ID number of IRCT 201101122709N16.

## Results

From 105 enrolled patients, 97 (31 in the EPA group, 33 in the DHA group and 33 in the control group) completed the study. There were 8 patients excluded from the study because of migration, adverse effects of drugs or supplements and refusing to continue. There were 12 males and 19 females in the EPA group with an average age of 35.54 ± 9.15 years old, 14 males and 19 females with an average age of 37.09 ± 10.28 years old in the DHA group, and 12 males and 21 females with an average age of 38.60 ± 10.92 years in the control group. There were no significant differences between the intervention and control groups regarding the distribution of sex, age, weight, daily dietary intake and the level of physical activity, at the beginning of the study; however, the body mass index (BMI) was significantly different (p=0.04). Moreover, no significant change was observed in all three groups in the mean weight, BMI, daily dietary intake and physical activity level during the study period (Tables 1, 2). In addition, there were no significant differences between the three groups in the levels of biochemical variables at the baseline (Table 3). The rate of eradication of infection in the three groups receiving EPA, DHA and placebo was not significantly different after the intervention, *i.e*. at the end of the study (Table 4).

According to the findings of the study, there was no significant difference between the three groups in the average levels of IL-6 and TAC, while the levels of IL-8 and hs-CRP were statistically different (p=0.03 and p=0.04, respectively). The results of post hoc tests showed significant differences between the patients receiving EPA and DHA supplement (Table 3).

In the EPA group, there was a significant reduction in the mean serum levels of IL-6, IL-8 and hs-CRP, at the end of the study as compared to the initial values (p=0.005, p=0.01 and p=0.002, respectively), while the change in the mean level of TAC was not significant. In the DHA group, there was a significant reduction in the mean serum levels of IL-6 and IL-8, at the end of the study as compared to the initial values (p=0.002 and p=0.01, respectively), whereas the levels of hs-CRP and TAC did not change significantly. Similar to the EPA and DHA groups, there was a significant reduction in the serum level of IL-6 (p=0.002) in the control group, as well (Table 3).

## Discussion

The present study was conducted to compare the effects of Eicosapentaenoic Acid and Docosahexaenoic Acid supplementation on H. pylori eradication, serum levels of some inflammatory markers and total antioxidant capacity.

Statistical analysis of Cochran’s & Mantel-Haenszel was applied as there was the likelihood that the significant difference between the three groups, in BMI, would have an influence, as a confounding variable. The results of this analysis suggested that the mean difference of the BMI of patients, in the three groups, could not be a confounding variable in the interpretation of the findings. 

As there is no evidence of previous studies on the effects of EPA and DHA supplements on H. pylori eradication, serum inflammatory markers and total antioxidant capacity in patients infected with this type of infection, the studies reporting findings similar to our study, were taken in to consideration. 

This study demonstrated that Eicosapentaenoic Acid or Docosahexaenoic Acid supplementation had no significant differential effect on the eradication of H. pylori infection in H. pylori positive patients. R. Meier *et al.* (2001) noticed that receiving a daily dosage of 1.5 g Eicosapen supplement instead of Metronidazole, along with Clarithromycin and Pantoprazole, in a three-drug regimen, for 7 days, was not useful in the eradication of the infection. This finding is consistent with the results of the present study. The findings also explained the cause of the effect of n-3 FAs on H. pylori eradication was the induction of metabolic enzymes of cytochrome P450 family by Eicosapen supplement resulting in reduction of plasma concentrations of drugs used to treat the infections. Several cytochrome P450 isoforms are responsible for metabolism of the drugs used by R. Meier in the eradication of the infection. Expression of cytochrome P450 2C19 enzyme has recently been shown to be effective in eradicating H. pylori (19). Some studies have mentioned that the minimal rate of eradication of the infection, after receiving polyunsaturated fatty acids (PUFAs), is due to inadequate dose of PUFA or short duration of treatment (20). Thompson *et al.* (1994) showed that Helicobacter incubation in micro-aerophilic conditions, with a range of PUFAs such as linoleic acid, arachidonic acid, linolenic acid (n-3), linolenic acid (n-6) and eicosapenaenoic acid, had an inhibitory effect on the growth of the bacteria. In that study, almost all microorganisms were killed by using linolenic acid concentration of 0.001 M while at lower concentrations of PUFAs, moving of H. pylori was inhibited. Nevertheless, the study did not show the inhibitory effect of PUFAs on the growth of H. pylori *in-vivo*, which was similar to our findings. When incubated with gram-negative bacteria in anaerobic culture *in-vitro*, PUFAs enter into the outer membrane of a gram-negative organism and significantly increase the fluidity of the membrane, which explains the positive effect of PUFAs on the growth inhibition of Helicobacter species. By opening the permeability channels between the organism and the environment, concentration gradient (*e.g.* for hydrogen ions) would lead to the disintegration of the organism and consequently it's death (21).

In the present study, it was shown that daily consumption of 2 g EPA and DHA capsules for 12 weeks did not appear to have a different effect on the mean level of IL-6. The average level of this marker decreased significantly in each of the three groups after the intervention period while this reduction was not significantly different in the three groups examined. Yet, the findings of this study showed that the mean level of serum IL-6, similar to the EPA and DHA group, reduced significantly in the control group. Several studies have shown that IL-6 serum level is high as long as H. pylori infection is present, and its concentration gradually reduces after the treatment of the infection (42,43). In our study, given that the control group as well as two other groups of patients had received medical protocol for eradication of infection, the observed phenomenon could be due to the effect of medical treatment on the level of the inflammatory factor. Moreover, EPA and DHA supplementation for 12 weeks at the dose of 2 g per day had a different effect on the mean serum level of IL-8. Although the average level of this indicator, in the three groups, reduced after the intervention period, this reduction was significant only in two groups receiving EPA and DHA supplements. EPA and DHA supplementation of 2 g daily for 12 weeks had a different effect on the mean serum level of hs-CRP, as well. The data from Freund-Levi *et al. *(2009) showed no effect of the consumption of 4 g daily of omega-3 supplements (containing EPA and DHA) for 6 months on plasma levels of IL-6 and hs-CRP in Alzheimer's patients (44). In that study, however, the dose and duration of supplementation were higher than those in our study. Moreover, the basic concentration of the patients' serum factors in that study was lower than that of our study while the observed reduction after the intervention was not statistically significant which might be due to the ineffectiveness of the intervention on the dependent variables (45). Contrary to the results of our study, Saifullah *et al.* (2007) suggested that the intake of daily 1.3 g EPA and DHA supplement in hemodialysis patients with the ratio of 2:1 for 12 weeks resulted in a significant reduction in serum CRP level. In that study, the basic concentration of the serum factors of the patients was much higher than that of the patients in our study, this may explain why despite the use of lower doses, the observed reduction in inflammatory factors was statistically significant, as the researchers believed that anti-inflammatory effects of n-3 PUFAs in individuals with higher baseline CRP level could be detected better (37). Similar to the findings of our study, Barbosa *et al.* (2010), reported that parenteral intake of a mixture of oils, including 6.4 g of n-3 FA for 5 days in septic patients, caused a significant reduction in serum IL-6 concentration (38). Due to the high dose of n-3 FA and very high basic concentration of IL-6 in septic patients compared to the patients of our study, significant results were observed; however, the intervention period in that study was much shorter than our study (45). The mechanisms by which n-3 FAs may inhibit pro-inflammatory cytokines production, have been frequently examined. The effects of n-3 PUFAs on inflammatory cytokines gene expression suggest that they might act in a way that modify the activity of transcription factors, most likely nuclear factor-κB (NF-κB) and/or peroxisome proliferator activated receptor (PPAR)-γ (46). There is a multidimensional relationship between NF-κB and pro-inflammatory cytokines. For example when the level of pro-inflammatory cytokine TNF-α or IL-1 elevates, it can heighten the NF-κB action, and then NF-κB affects positively on the expression of cytokines like TNF-α itself and also IL-6, IL-8 and others; so the inflammatory process exacerbates (47). Acting in an anti-inflammatory manner and directly regulating inflammatory gene expression, PPAR-γ interferes with the activation of NF-κB (46), which is thought to have a pivotal role in immune and inflammatory responses through the regulation of genes encoding pro-inflammatory cytokines, adhesion molecules, chemokines, growth factors and inducible enzymes, such as COX2 and inducible nitric oxide synthase (iNOS) (27). Omega-3 fatty acids can bind to the PPAR-α and PPAR-γ, which regulate the transcription of target genes. PPARs can also repress gene transcription by interfering with signaling molecules, such as NF-κB, thereby inhibiting the production of pro-inflammatory cytokines (18). The studies demonstrating the effects of omega-3 on serum inflammatory markers, have often mentioned these mechanisms. 

The present study has shown that EPA and DHA supplements, 2 g daily for 12 weeks, did not show to have different effects on the mean level of TAC. The study of Toorang *et al*. (2009) showed daily intakes of 2714 mg n-3 FAs (1548 mg EPA and 828 mg DHA) for 8 weeks had no effect on the serum total antioxidant capacity in patients with type II diabetes mellitus (48). This finding is consistent with the results of our study. Sarbolouki *et al.* (2010) in the intervention practice, found a significant effect of supplementation with 3 g EPA for 3 months on total antioxidant capacity and antioxidant enzymes in diabetic patients (49). This is also in line with the findings of our study. Moreover, Mahdavi *et al.* (2011) in a clinical trial discovered that 3 g of supplement containing 1.8 g EPA and 1.2 g DHA for 1.5 months increased serum level of total antioxidant capacity and prevented the enhancement of oxidative stress in patients with gastric cancer undergoing chemotherapy (50).

Although, the differences in the results of aforementioned studies may be due to different methods used to measure this indicator, it should be noted that concentration of PUFAs, particularly EPA, is a risk factor in the development of oxidative stress (48).

In conclusion, present study showed that EPA and DHA supplementation at a dose of 2 g daily for 12 weeks in patients infected with H. pylori, had no differential effects on the eradication of H. pylori, mean serum levels of IL-6 and TAC, while it appeared to have different effects on the levels of IL-8 and hs-CRP.

The present study is the first of its kind, aimed to compare the effects of EPA and DHA on the eradication of H. pylori, serum concentration of some anti-inflammatory cytokines and total antioxidant capacity in infected patients, whose information regarding potential confounding variables, including dietary intake and physical activity level, were available. However, this study had limitations, as well. First, several patients participating in the study, were excluded from the study. The second limitation was lack of plasma level measurements of n-3 FAs for a better monitoring of the patients’ compliance. Moreover, frequent inclusion criteria, especially having no metabolic diseases, taking no supplements of omega-3 and antioxidant supplement and no previous history of infection, led to further reduction in the number of patients eligible for the study and hampered the disease finding process with many difficulties. 

Finally, it is suggested to conduct similar studies with higher number of patients and longer study period for a better observation of the effects of inflammatory factors. Another research study should be conducted with different doses of n-3 FAs to investigate dose-related effects of n-3 FAs on inflammatory markers and plasma concentration measurement of n-3 FAs, after the supplement intake, to ensure the compliance rate of supplementation and effective dose of absorption which would explain the mechanism of the compounds.
